# Delivery and payment reform in Massachusetts: substance use disorder treatment organizations’ perspectives

**DOI:** 10.1186/1940-0640-10-S1-A51

**Published:** 2015-02-20

**Authors:** Amity E Quinn, Connie M Horgan, Mary Brolin, Maureen T Stewart, Dominic Hodgkin, Nancy Lane

**Affiliations:** 1 Heller School for Social Policy and Management, Brandeis University, Waltham, MA, 02454, USA; 2 Massachusetts Behavioral Health Partnership, Boston, MA, 02118, USA

## Background

The Affordable Care Act and many states’ health-care reforms present opportunities and challenges for the substance use disorder (SUD) treatment system. These reforms foster the implementation of new payment and delivery system models that emphasize care coordination and make providers more responsible for patients’ clinical management and costs. Bundled payments, a predetermined fee or budget that includes the prices of a group of services that would typically treat an episode of care in a defined period of time, are considered one of the most promising new payment models. Bundled payments for SUD treatment have the potential to improve care coordination, implementation of evidence-based practices, and engagement in outpatient treatment; reduce readmissions to detox; make care more patient-centered; and cover treatment services that are not traditionally reimbursed. The objective of this study was to obtain SUD treatment organizations’ perspectives on health-care reform and the design of bundled payments for SUD treatment.

## Methods

Organizational interviews were conducted with eight SUD treatment organizations in Massachusetts, with a total of 22 executive, clinical, and financial leaders. Organizations were selected using a purposive sampling procedure. Thirty- to 90-minute interviews were conducted with 1–4 participants at each of the three organization types of interest: a) detoxification services only (n = 1); b) detoxification and outpatient services (n = 4); and c) outpatient services only (n = 3). Interviews addressed the challenges and opportunities of Federal and state health reforms and the design of SUD bundled payments, including patients’ paths through the treatment system, which services should be included, and the length of a treatment episode. Data were analyzed using a framework analysis approach, an ideal analysis method for policy research.

## Results

Preliminary themes emerging from providers are concerns about low reimbursement rates, how to integrate with the general health-care system, the need to improve outpatient treatment engagement, facilitating the relationship between detox and outpatient providers, and concerns about financial accountability.

## Conclusions

Bringing SUD treatment organizations’ perspectives into the conversation about the role of SUD treatment in health-care delivery and financing transformations is critical to understanding the context of the health-care system in Massachusetts. The results of this study provide important information about how the SUD treatment system is responding to health-care reform and about the design and feasibility of bundled payments for SUD treatment. The results will also be used to design bundled payments for SUD treatment using claims and administrative data. These will be the first bundled payments for SUD treatment and have the potential to be used in health-care practice and in further research to improve the health and health care of individuals with alcohol use disorders.

